# The Global Virus Network’s testimony on Ebola to the U.S. Senate

**DOI:** 10.12688/f1000research.5954.1

**Published:** 2014-12-15

**Authors:** Robert C. Gallo

**Affiliations:** 1 Co-Founder and Scientific Director, Global Virus Network (GVN), and Director, Institute of Human Virology, University of Maryland School of Medicine, College Park, MD, USA

## Abstract

In this Editorial I briefly outline the Global Virus Network’s testimony to the recent U.S. Senate’s “Fighting Ebola and Protecting America” committee hearing.

## Editorial

This past November, the Global Virus Network (GVN) was invited by the U.S. Senate Committee on Appropriations to submit testimony at a hearing on “U.S. Government Response: Fighting Ebola and Protecting America” ^1^. In our testimony, my colleagues and I outlined several propositions, some of which were drawn from my personal experiences in the early and subsequent days of the AIDS epidemic. We recommended expanded resources for basic science research to accelerate answers to questions about the source of the Ebola outbreak, human-to-human transmission modes, mutations and vaccines, among other necessary endeavors. We noted the importance of the U.S. government leveraging previous successes to advance translation of basic science discoveries into products that could be distributed across the globe as appropriate. It was important to highlight in the testimony that some of the important tools we are lacking in the current Ebola outbreak include proper diagnostics to detect virus infection before individuals are symptomatic. We noted the importance of science-driven communications as another important tool in disease prevention, and offered to host webinars to educate journalists reporting on the outbreak. Last, but certainly not least, it is important to expand medical virology training programs in affected nations to promote broad skills in detection and analysis of an array of viruses. Further, education and training in biosafety laboratory practices and the handling of blood products is important and lacking in Ebola-stricken West African nations.

The GVN supports *F1000Research*’s Ebola Collection initiative that enables all Ebola-related knowledge to be made available on a single open access platform within days of submission, including prioritization of those accepted and published free of charge. This initiative aligns with the GVN’s mission to advance research on all viral diseases, including those with pandemic potential; to train the next generation of medical virologists; to educate the public on viral threats and scientific responses; and to advocate for support for the medical virology field. The GVN is a coalition of leading medical virologists based in 31 GVN Centers of Excellence in 25 nations, including the U.S., India, China, Sweden, Germany, Italy, South Africa, the U.K., Argentina and Russia -- and it is growing. For our complete testimony, please click here ^2^.
